# Inflammatory Biomarkers Are Associated with Pain and Functional Impairment in Knee Osteoarthritis Patients: A Cross-Sectional Pilot Study

**DOI:** 10.3390/jcm15093466

**Published:** 2026-05-01

**Authors:** Luca Gallelli, Vincenzo Rania, Roberta Macrì, Mirza Suljagic, Andzelika Michniewicz, Daria Ler, Gianmarco Marcianò, Cristina Vocca, Caterina Palleria, Domenica Scumaci, Diana Marisol Abrego-Guandique, Maria Cristina Caroleo, Erika Cione

**Affiliations:** 1 Department of Health Science, University of Catanzaro, 88100 Catanzaro, Italy; roberta.macri@unicz.it (R.M.); gianmarco.marciano3@gmail.com (G.M.); cristina_vocca@live.it (C.V.); dianamarisol.abregoguandique@unicz.it (D.M.A.-G.); mariacristina.caroleo@unicz.it (M.C.C.); 2 Operative Unit of Clinical Pharmacology and Pharmacovigilance, “R Dulbecco” University Hospital, Viale Europa, 88100 Catanzaro, Italy; raniavincenzo1@gmail.com (V.R.); angymich@tiscali.it (A.M.); palleria@unicz.it (C.P.); 3 Research Center FAS@UMG (Farmacologia Applicata e di Sistema@Università Magna Graecia), Department of Health Science, University Magna Graecia, Viale Europa, 88100 Catanzaro, Italy; 4 Department of Molecular Laboratory Diagnostic, Sarajevo School of Science and Technology, Hrasnička Cesta 3a, 71210 Ilidža, Bosnia and Herzegovina; mirza@fablab.ba (M.S.); daria.ler@asainstitut.ba (D.L.); 5 Research Center on Advanced Biochemistry and Molecular Biology, Department of Experimental and Clinical Medicine, Magna Græcia University of Catanzaro, Viale Europa, 88100 Catanzaro, Italy; scumaci@unicz.it; 6 Research Center CRUISE (Clinical Research Unit for Innovation, Support and Ethics), Department of Health Science, University Magna Graecia, 88100 Catanzaro, Italy; 7 Department of Pharmacy, Health and Nutritional Sciences, University of Calabria, 87036 Rende, Italy; erika.cione@unical.it

**Keywords:** knee osteoarthritis, inflammatory biomarkers, cytokines, pain, systemic inflammation, mechanoflammation

## Abstract

**Background**: Osteoarthritis (OA) is a heterogeneous joint disorder traditionally considered mechanically driven; however, evidence indicates that inflammatory mechanisms contribute to symptom expression. Exploratory analyses of peripheral biomarkers may provide insights into systemic inflammation in symptomatic knee OA, but formal phenotypic validation requires dedicated clustering or longitudinal studies. **Objective**: To examine associations between clinical pain, functional impairment, and circulating inflammatory biomarkers in patients with knee OA compared with healthy controls. **Methods**: In this prospective, single-center study, patients aged 40–80 years with radiographically confirmed knee OA and chronic knee pain were compared with age- and sex-matched healthy controls. Pain intensity and functional status were assessed using the Visual Analogue Scale (VAS) and the Knee Injury and Osteoarthritis Outcome Score (KOOS). Circulating inflammatory biomarkers, including cytokines and matrix metalloproteinases, were quantified using multiplex immunoassays. Statistical analyses included adjusted linear regression models, with age and BMI as covariates, and multiple testing correction using the Benjamini–Hochberg procedure (FDR alpha error 5%). **Results**: OA patients exhibited higher circulating levels of TNF-α, IL-6, IL-8, MMP-1, MMP-3, TNFSF13, TNFSF13B, and pentraxin-3 compared with controls (*p* < 0.01). No significant sex differences were observed. KOOSs correlated with IL-6 and IL-10 levels, suggesting an association between systemic inflammatory activity and functional limitation. All findings are presented as exploratory and associative. **Conclusions**: Patients with knee OA display systemic inflammatory biomarker differences associated with pain and functional impairment. These results support the role of inflammation in OA symptoms within an exploratory framework. Larger, longitudinal studies are warranted to validate these observations.

## 1. Introduction

Osteoarthritis (OA) is the most common degenerative joint disorder and a leading cause of pain and disability worldwide. Although it can affect any synovial joint, the knee is the most frequently involved and clinically burdensome site. Patients typically present with chronic joint pain, stiffness, functional limitations, and reduced quality of life [1]. Despite its high prevalence, OA remains a heterogeneous condition, with considerable variability in symptom severity, structural damage, and disease progression [2,3]. This heterogeneity reflects a complex pathophysiology involving genetic predisposition, metabolic abnormalities, biomechanical stress, and environmental risk factors. Historically, OA was considered a purely mechanical “wear-and-tear” disease [4], with inflammatory features regarded as secondary or clinically insignificant [5]. However, growing evidence indicates that inflammation is present in a substantial proportion of patients with OA and contributes directly to symptoms and disease progression. Inflammatory activity may be localized within the joint, manifesting as synovitis or periarticular inflammation, or detectable systemically. The interplay between mechanical stress and inflammation—termed mechanoflammation—is now recognized as a key component of OA pathology and symptom expression [6]. Knee OA is characterized by progressive cartilage loss, osteophyte formation, subchondral bone remodeling and sclerosis, and, in advanced stages, subchondral cyst formation [7]. Pain in knee OA cannot be fully explained by structural damage alone. Instead, it reflects the combined effects of local inflammation, altered bone and cartilage metabolism, release of inflammatory mediators, and sensitization of peripheral and central pain pathways [1,8]. These mechanisms contribute to the frequently observed poor correlation between radiographic severity and patient-reported pain. Circulating inflammatory biomarkers, such as interleukin-6 (IL-6) and tumor necrosis factor-α (TNF-α), have been associated with both radiographic disease severity and symptom burden in OA patients [9,10]. An imbalance in cytokine signaling favoring pro-inflammatory mediators promotes a catabolic intra-articular environment via activation of matrix metalloproteinases (MMPs), leading to progressive cartilage degradation and joint tissue damage [11]. Although individual inflammatory mediators have been extensively studied in OA, there remains limited clinically contextualized evidence linking coordinated systemic inflammatory profiles to validated patient-reported pain and functional outcomes in well-characterized populations with symptomatic knee OA. This gap highlights the need for integrative analyses that combine multiplex biomarker assessment with standardized clinical measures within clearly defined cohorts. While the roles of cytokines, such as TNF-α, IL-6, IL-8, and MMPs in OA are well established, fewer studies have examined their coordinated systemic expression in symptomatically well-characterized populations using validated patient-reported outcome measures. Given the increasing recognition of inflammation as a clinically relevant component of OA, there is a growing need for biomarkers capable of identifying patients with distinct inflammatory patterns associated with pain, thereby guiding personalized management strategies. The novelty of the present study does not lie in the identification of new molecular mediators, but in the translational integration of multiplex biomarker profiling with validated symptom-based instruments, such as the Visual Analogue Scale (VAS) and the Knee Injury and Osteoarthritis Outcome Score (KOOS), emphasizing symptom-driven characterization rather than structural severity alone. Accordingly, this exploratory observational study evaluated clinical parameters alongside peripheral blood biomarkers to examine their associations with pain and functional impairment in patients with knee OA, compared with healthy controls.

## 2. Materials and Methods

### 2.1. Study Design

This investigation was designed as a cross-sectional exploratory pilot study conducted within a prospective, single-center observational framework. The study aimed to evaluate systemic inflammatory biomarker profiles in patients with symptomatic knee osteoarthritis (OA) and to explore their association with validated clinical measures of pain and functional impairment.

### 2.2. Participants

Consecutive patients aged ≥18 years with symptomatic knee OA were recruited at the Pain Unit of the Clinical Pharmacology and Pharmacovigilance Department, “R. Dulbecco” University Hospital of Catanzaro, Italy, between January and November 2025 (Institutional Ethics Committee approval no. 120/2018; Clinical Trial Registration: NCT05509075).

An age- and sex-matched control group of healthy volunteers without knee pain or radiographic evidence of OA was enrolled. Comparative analyses between OA patients and healthy controls are presented in Results Section 3.1 (see also Tables S1 and S2), to highlight differences in systemic inflammatory biomarker levels. All participants provided written informed consent.

**Figure 1:**
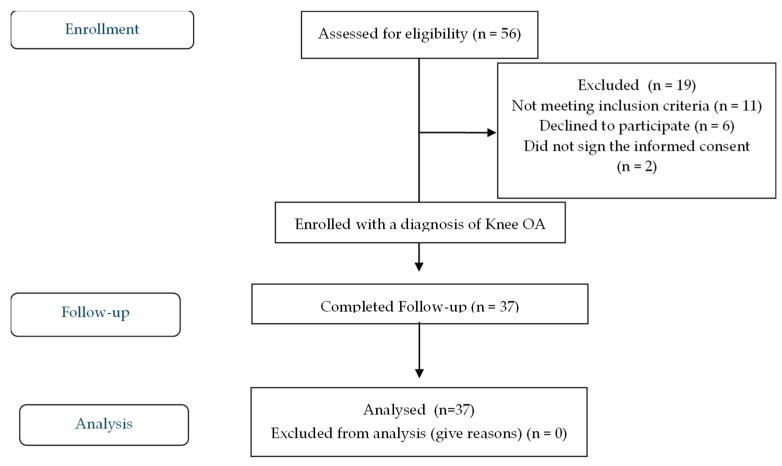
Strengthening the Reporting of Observational Studies in Epidemiology (STROBE) flow diagram of patient enrollment and study selection.

The study was conducted in accordance with the Declaration of Helsinki, Good Clinical Practice guidelines, and national privacy regulations. Reporting adhered to the STROBE (Strengthening the Reporting of Observational Studies in Epidemiology) guidelines. A participant selection flow diagram is provided in Figure 1.

### 2.3. Sample Size and Power

Sample size was determined as a priori using TNF-alpha as the reference biomarker, assuming a medium effect size (Cohen’s d = 0.6), alpha = 0.05, and 80% power, requiring ≥30 participants per group. To account for possible attrition, the target enrollment was increased to 33 subjects per group. IL-6 was also considered based on comparable biological relevance. Analyses of additional biomarkers were exploratory; the study was not powered for multiple secondary endpoints. A total of 98 individuals were initially screened for eligibility.

### 2.4. Inclusion and Exclusion Criteria

Patients were eligible if they were aged ≥18 years, had radiographic evidence of knee OA graded 1–4 according to the Kellgren–Lawrence scale, reported chronic knee pain for at least three months with a Visual Analogue Scale (VAS) score ≥ 4, and were able to provide written informed consent and complete all clinical assessments. Although additional structural features (e.g., meniscal tears, synovial burden, inflammatory cell composition, and calcium pyrophosphate deposits) may influence symptom severity and cytokine levels, MRI evaluation was not performed, as it was beyond the scope of this exploratory pilot study. Exclusion criteria included inflammatory or autoimmune rheumatic diseases, advanced malignancy, osteoporosis, current or recent (within four weeks) use of systemic nonsteroidal anti-inflammatory drugs or corticosteroids, recent intra-articular injections, known autoimmune disorders, or any clearly defined medical condition known to significantly influence systemic inflammatory biomarker levels (e.g., acute infection or systemic inflammatory conditions). Detailed reasons for exclusion and the final number of participants included in each group are presented in the selection flow diagram (Figure 1).

### 2.5. Experimental Protocol

#### 2.5.1. Clinical and Radiographic Assessment

At baseline, demographic data, comorbidities, medication history, and lifestyle factors were recorded. Pain intensity was assessed using VAS [12,13], while knee-related symptoms and functional impairment were evaluated using KOOS [12,13,14].

Standard anteroposterior and lateral knee radiographs were obtained for all patients. Disease severity was graded according to the Kellgren–Lawrence scale by two independent radiologists blinded to clinical and biomarker data. Inter- and intra-observer reliability was excellent (ICC = 0.98 and 0.96, respectively).

#### 2.5.2. Biomarker Collection and Analysis

Peripheral venous blood samples were collected under standardized conditions. Blood sampling was performed in the morning after an overnight fast of at least 8 h to minimize biological variability. Serum was separated and stored at −80 °C until analysis.

Circulating inflammatory biomarkers were quantified using magnetic bead–based multiplex immunoassays (Bio-Plex^®^, Bio-Rad Laboratories, Milan, Italy), allowing simultaneous measurement of multiple inflammatory mediators. All assays were performed duplicate according to the manufacturer’s instructions.

The selected panel was designed to capture key inflammatory and immunological pathways implicated in osteoarthritis-related pain, including pro-inflammatory signaling, anti-inflammatory regulation, and tissue remodeling processes. Pro-inflammatory cytokines such as TNF-α, IL-6, and IL-8 have been associated with pain severity, disease progression, and functional impairment in patients with osteoarthritis [9,10,15,16].

Matrix metalloproteinases (MMP-1 and MMP-3) were selected due to their established role in cartilage degradation and joint structural damage [17,18].

TNFSF13 (APRIL) and TNFSF13B (BAFF) were included for their involvement in B-cell activation and immune regulation, which are increasingly recognized as contributors to chronic low-grade inflammation [19,20].

Pentraxin-3 was included as a marker of innate immune activation and tissue remodeling, with emerging evidence supporting its role in inflammatory joint conditions [21].

Overall, this panel was selected to provide a targeted, yet comprehensive assessment of inflammatory pathways potentially associated with pain and functional outcomes in osteoarthritis.

### 2.6. Outcome Measures

Pain intensity was assessed using the VAS, a 10 cm horizontal line anchored at 0 (“no pain”) and 10 (“worst pain imaginable”), on which participants indicated their current level of knee pain. Knee-related symptoms, functional limitations, and health-related quality of life were evaluated using the KOOS, a validated, disease-specific instrument assessing five domains: pain, other symptoms, activities of daily living, sport and recreation function, and knee-related quality of life. Each subscale is scored from 0 to 100, with higher scores indicating better knee function and lower symptom burden.

### 2.7. Endpoints

The primary endpoint was to evaluate differences in circulating TNF-α and IL-6 levels between patients with knee OA and healthy controls, based on evidence linking these cytokines to pain severity in knee OA [22,23]. Secondary endpoints included the assessment of associations between biomarker concentrations and clinical and functional parameters (VAS and KOOS), exploring the relationship between systemic inflammation, pain severity, and functional impairment.

### 2.8. Statistical Analysis

Continuous variables were first assessed for normality using the Shapiro–Wilk and Kolmogorov–Smirnov tests. Normally distributed variables are reported as mean ± standard deviation (SD), while non-normally distributed variables are summarized as median and interquartile range (IQR). Categorical variables are expressed as counts and percentages.

Between-group comparisons for continuous variables were conducted using Student’s *t*-test or one-way ANOVA when parametric assumptions were met; for non-normally distributed variables, Mann–Whitney U or Kruskal–Wallis tests were applied. Categorical variables were analyzed using the chi-square test or Fisher’s exact test as appropriate. For all main analyses, 95% confidence intervals (CI) were calculated to provide precision estimates for mean differences and effect sizes. Cohen’s d was reported for standardized effect size assessment.

Multivariate linear regression models were constructed to adjust for potential confounders, including age, sex, BMI, smoking status, alcohol consumption, physical activity, comorbidities, and concomitant medications. Biomarkers were analyzed as dependent variables in separate models, and clinical outcomes (VAS and KOOSs) were included as independent variables. Logarithmic transformation was applied to skewed biomarkers to meet model assumptions. Model selection was guided by clinical relevance and previous literature, while recognizing that the small sample size limits the inclusion of many covariates.

To account for multiple testing across the biomarker panel, the False Discovery Rate (FDR) was controlled using the Benjamini–Hochberg procedure with an alpha threshold of 0.05. Post hoc analyses were performed using adjusted *p*-values where appropriate. Correlations between biomarkers and clinical outcomes were assessed using Pearson or Spearman correlation coefficients depending on the distribution of the variables.

Repeated measures were analyzed using the Friedman test followed by Wilcoxon signed-rank tests for pairwise comparisons. All statistical tests were two-tailed, and a *p*-value < 0.05 was considered statistically significant unless adjusted for multiple comparisons. All analyses were performed using SPSS version 22.0 (IBM Corp., Armonk, NY, USA). No unsupervised clustering, principal component analysis, or formal inflammatory phenotype operationalization was performed; thus, all interpretations are strictly exploratory.

## 3. Results

### 3.1. Study Population

A total of 56 patients with knee OA were initially screened, of whom 37 (22 women, 15 men) met all inclusion criteria and provided informed consent (Figure 1; Table 1) (see also Table S1). Fifty healthy control subjects (28 women and 22 men) were recruited to match the OA group for age, sex, and BMI. No statistically significant differences were observed between groups for these characteristics (*p* > 0.05).

The mean age of control group patients was 62.25 ± 9.8 years, with no significant difference between women (61.03 ± 9.2) and men (63.04 ± 10.5, 9) (*p* = 0.48; effect size: 0.065). Mean BMI (26.5 ± 3.3) was similar between sexes (women 26.4 ± 3.1, men 25.7 ± 1.9; *p* = 0.2, effect size 0.18).

Demographic and clinical characteristics of knee OA patients are summarized in Table 1. The mean age of OA patients was 62.5 ± 12.4 years (95% CI: 58.4–66.7), with no significant difference between women (61.2 ± 12.5, 95% CI: 55.8–66.6) and men (64.7 ± 12.5, 95% CI: 57.5–71.9). Mean BMI was similar between sexes (women 27.4 ± 3.7, men 26.0 ± 2.6). Overall, OA patients reported clinically relevant pain and functional limitation, as reflected by mean VAS scores of 7.4 ± 0.6 and KOOSs of 45.9 ± 4.5.

In knee OA patients, correlation analysis with Bonferroni correction did not reveal any significant correlations between pain, BMI, and age (Table 2). In contrast, age was inversely associated with KOOSs (r = −0.454; *p* = 0.005), reflecting increased functional impairment with advancing age. No significant correlations were observed between KOOS and BMI (r = 0.300; *p* = 0.071), smoking status (r = −0.095; *p* = 0.578), or diabetes (r = −0.056; *p* = 0.741) in either sex. Additionally, pain intensity (VAS) was not significantly correlated with age or BMI in either sex.

### 3.2. Biomarkers Evaluation

Patients with OA exhibited significantly higher circulating levels of matrix-degrading enzymes (MMP-1, MMP-3, TNFSF13, TNFSF13B, and pentraxin-3) and pro-inflammatory cytokines (TNF-α, IL-6, IL-8) compared with healthy controls (TNFSF13: 1781.18 ± 160.1 pg/mL; TNFSF13B: 1719.41 ± 144.6 pg/mL; MMP-1: 831.7 ± 211.3 pg/mL; MMP3: 5291.3 ± 1361.4 pg/mL; Pentraxin-3: 619.9 ± 87.3 pg/mL; TNF-alpha: 3.8 ± 1.8 pg/mL; IL-6: 1.63 ± 0.8 pg/mL; IL-8: 16.7 ± 5.1 pg/mL; IL-10: 2.5 ± 1.3 pg/mL. *p* < 0.01) (see Supplementary Data). One-way ANOVA did not identify significant differences among knee OA patients (*p* > 0.05; Table 3), and no sex-related differences were observed for any of the evaluated cytokines (Table 4 and Table 5) (see Tables S1 and S2). Similarly, Kruskal–Wallis testing did not reveal significant differences in cytokine distributions between men and women with pain (MMP-3: men *p* = 0.269, women *p* = 0.830; MMP-1: men *p* = 0.783, women *p* = 0.847; IL-6: men *p* = 0.265, women *p* = 0.511; IL-8: men *p* = 0.415, women *p* = 0.112; IL-10: men *p* = 0.144, women *p* = 0.885; TNF-α: men *p* = 0.222, women *p* = 0.544; pentraxin-3: men *p* = 0.199, women *p* = 0.451; TNFSF13: men *p* = 0.061, women *p* = 0.857; TNFSF13B: men *p* = 0.230, women *p* = 0.548).

Correlation analyses revealed biologically coherent relationships among biomarkers. TNF-α and IL-6 were strongly positively correlated (r = 0.72, *p* < 0.001), highlighting their coordinated role in inflammatory signaling. TNFSF13B correlated with TNF-α (r = 0.68, *p* < 0.001), IL-6 (r = 0.63, *p* < 0.001), and MMP-1 (r = 0.41, *p* = 0.04), while TNFSF13 correlated with pentraxin-3 (r = 0.35, *p* = 0.027). These associations suggest the existence of an interconnected immune–catabolic network in symptomatic OA.

Analyses linking biomarkers to clinical outcomes demonstrated that KOOSs were inversely associated with IL-6 (r = −0.337; 95% CI: −0.60 to −0.03; *p* = 0.041) and positively associated with IL-10 (r = 0.449; 95% CI: 0.15–0.69; *p* = 0.005) (Table 6). After controlling multiple comparisons using the Benjamini–Hochberg FDR method (alpha error = 0.05), the correlation with IL-10 remained significant, suggesting that higher anti-inflammatory signaling may partially counterbalance functional impairment. No significant correlations were observed between VAS scores and individual biomarkers (TNFSF13: *p* = 0.711; TNFSF13B: *p* = 0.576; MMP1: *p* = 0.889; MMP3: *p* = 0.448; Pentraxin3: *p* = 0.785; TNF-alpha: *p* = 0.664; IL-6: *p* = 0.461; IL-8: *p* = 0.091; IL-10: *p* = 0.777), indicating that functional limitation may be more closely linked to systemic inflammatory activity than current pain intensity.

Multivariate linear regression models adjusted for age, sex, BMI, smoking status, alcohol consumption, physical activity, comorbidities, and concomitant medications confirmed that IL-6 and IL-10 were independently associated with KOOSs (adjusted β = −0.31, *p* = 0.048 for IL-6; adjusted β = 0.42, *p* = 0.007 for IL-10). Log-transformation of skewed biomarkers ensured model assumptions were met, and 95% confidence intervals provide precision estimates for effect sizes.

Overall, these findings indicate that patients with knee OA exhibit a consistent systemic inflammatory profile, independent of sex, which is associated with functional limitation. The data supports the hypothesis that multiplex biomarker profiling can complement clinical assessment, providing insight into symptom severity and the underlying inflammatory milieu.

## 4. Discussion

In this study, we explored the relationship between systemic inflammatory biomarkers and clinical outcomes in patients with symptomatic knee osteoarthritis (OA), compared with age- and sex-matched healthy controls. Our findings indicate that inflammatory processes are associated with OA pathophysiology even in patients without overt systemic inflammatory disease, highlighting that, due to the cross-sectional and exploratory design, these associations are descriptive rather than causal. They reinforce the contemporary view of OA as a condition shaped by the dynamic interplay between biomechanical stress and inflammatory mechanisms, rather than as a purely degenerative or mechanical disorder [2,6].

In line with previous studies reporting elevated inflammatory cytokines in OA [10], our cohort exhibited significantly increased circulating levels of pro-inflammatory cytokines (TNF-α, IL-6, IL-8), matrix metalloproteinases (MMP-1, MMP-3), and immune-regulatory mediators (TNFSF13, TNFSF13B, pentraxin-3), compared with age- and sex-matched healthy controls as detailed in Section 3. These observations are consistent with prior reports linking systemic inflammatory markers to radiographic severity, cartilage loss, and disease progression [9], supporting the association between inflammatory-catabolic pathways and the clinical expression of knee OA [11,24].

Thus, our findings largely confirm existing literature while reinforcing these associations within a clinically characterized cohort.

The elevated levels of pentraxin-3 observed in our OA patients further support the presence of low-grade systemic inflammation. Pentraxin-3 is a key mediator of innate immunity, tissue remodeling, and chronic inflammatory processes, and has been implicated in musculoskeletal and inflammatory conditions independent of metabolic syndrome [25,26]. Its increased concentration suggests that inflammatory activation in knee OA extends beyond the joint and may contribute to pain and functional impairment, with the inclusion of pentraxin-3 alongside TNFSF13 and TNFSF13B representing a novel exploratory contribution of this study.

The absence of significant sex differences in biomarker concentrations suggests that systemic inflammation represents a disease-driven characteristic of symptomatic knee osteoarthritis rather than a sex-specific phenomenon. This finding contrasts with previous literature describing sex-related differences in pain perception and immune response [8], yet it underscores that, within this cohort, the systemic inflammatory profile appears to be primarily associated with disease activity. Overall, these findings suggest a broadly consistent systemic inflammatory biomarker profile among patients with clinically relevant pain, although further stratified analyses (e.g., by OA grade or pain severity) are needed to confirm this observation. However, no formal clustering or phenotypic validation was performed; therefore, our results describe a common inflammatory profile rather than a validated inflammatory phenotype.

Correlation analyses revealed biologically plausible interactions among key mediators. The strong association between TNF-α and IL-6 underscores their central role in coordinating inflammatory signaling in OA [11]. TNF-α and IL-6 were strongly positively correlated (*p* < 0.001), independent of sex and age. Additionally, TNFSF13B was significantly correlated with TNF-α, IL-6, and MMP-1, while TNFSF13 correlated with pentraxin-3 (*p* = 0.027), suggesting an interconnected immune–catabolic network linking cytokine activation to extracellular matrix remodeling. These relationships align with experimental evidence showing that pro-inflammatory cytokines directly induce MMP expression in chondrocytes, accelerating cartilage breakdown and contributing to structural joint damage [7,11]. While these mechanisms are well established experimentally, the present study provides associative clinical support within a symptomatic OA population.

In parallel, our results highlight a clinically meaningful link between systemic inflammation and patient-reported outcomes. KOOSs correlated with circulating IL-6 and IL-10, reflecting the association between systemic inflammation and pain severity as well as functional limitation. IL-6 has been implicated in peripheral nociceptor sensitization and central pain amplification [1,8], providing a mechanistic explanation for chronic pain in knee OA, while the correlation with IL-10, after FDR correction, may indicate a compensatory anti-inflammatory response counterbalancing persistent pro-inflammatory signaling. Integrating multiplex biomarker assessment with validated functional outcome measures represents a key contribution of this exploratory investigation.

Collectively, these findings support the mechanoflammation paradigm [6], whereby mechanical loading and inflammatory signaling jointly drive OA onset and progression. Elevated pentraxin-3 levels in our OA cohort indicate low-grade systemic inflammation beyond the joint, potentially contributing to pain and functional limitation. The integration of immune-regulatory biomarkers (TNFSF13 and TNFSF13B) alongside classical pro-inflammatory mediators represents a novel exploratory contribution, illustrating a broader network of systemic inflammatory activity in symptomatic OA.

Nevertheless, results must be interpreted within the limits of a cross-sectional pilot study, which cannot establish causality. From a clinical perspective, identifying an inflammatory OA profile via peripheral blood biomarkers may inform patient stratification, prognostic refinement, and targeted therapeutic approaches. At this stage, our findings characterize an inflammatory biomarker pattern associated with symptomatic knee OA rather than validating a clinically applicable stratification model. Circulating biomarkers, as accessible and minimally invasive tools, may complement clinical assessment and imaging in routine practice.

This study has several limitations. The use of the Kellgren–Lawrence scale alone may not fully capture structural and inflammatory joint alterations that could influence pain and biomarker profiles. Advanced imaging techniques, such as MRI, may allow a more comprehensive characterization and should be considered in future studies. In addition, the cross-sectional, single-center design, based on a single time-point assessment, precludes evaluation of intra-individual variability in circulating inflammatory biomarkers and limits causal inference. Cytokine levels are known to fluctuate over time, and therefore the present findings should be interpreted with caution, as they do not capture temporal dynamics. Although consecutive patients were enrolled, recruitment from a single tertiary-care Pain Unit may have introduced referral bias, as individuals evaluated in specialized hospital settings often present with more severe, treatment-resistant, or clinically complex disease compared with community-based populations. Consequently, the findings may not be fully representative of the broader knee osteoarthritis population, limiting their external validity. Future longitudinal studies with repeated measurements are warranted to better characterize the temporal variability of inflammatory biomarkers and their relationship with clinical outcomes.

The relatively small sample size restricts statistical power, particularly for secondary biomarkers, and precludes extensive multivariate modeling. Moreover, the analysis of multiple circulating biomarkers increases the risk of type I error. Although multiple comparisons were addressed using the Benjamini–Hochberg procedure to control the FDR at 5%, residual inflation of false-positive findings cannot be entirely excluded, especially given the exploratory nature of the study.

Multivariate linear regression models were adjusted for clinically relevant confounders; however, residual confounding due to unmeasured or imprecisely measured variables (e.g., dietary factors, subclinical inflammatory conditions, medication adherence, or genetic predisposition) may persist. The limited sample size also constrained the number of covariates that could be included simultaneously without overfitting, potentially affecting the stability of the regression estimates. Circulating biomarkers may not fully reflect local joint inflammation, although previous evidence suggests systemic cytokine levels can mirror disease-relevant processes in OA [9]

. Moreover, radiographic severity was not correlated with biomarker levels, and no unsupervised clustering or formal phenotypic validation was performed; therefore, the observed patterns describe associative inflammatory profiles rather than validated phenotypes or clinically actionable stratifications. Overall, the results should be interpreted as hypothesis-generating rather than confirmation. Despite these limitations, this study has notable strengths. Participants were well-characterized clinically, with standardized assessment of pain and function using validated instruments (VAS and KOOS). A well-matched healthy control group enabled comparative evaluation of systemic biomarker levels. Multiplex immunoassays allowed simultaneous quantification of multiple cytokines, MMPs, and immune-regulatory mediators, providing an integrative view of systemic inflammatory activity.

In conclusion, patients with knee OA exhibit a systemic inflammatory biomarker profile associated with functional impairment, independent of sex. These results suggest that peripheral biomarker profiling may offer complementary information to clinical evaluation and provide exploratory insights into the inflammatory mechanisms underlying OA-related symptoms. However, no formal phenotypic classification or patient stratification model was validated; therefore, these findings should be considered hypothesis-generating. Larger, longitudinal studies are needed to confirm these observations, clarify temporal relationships, and assess their potential relevance for personalized therapeutic approaches.

## Figures and Tables

**Table 1:** Demographic and clinical characteristics of the study population with knee OA (n = 37). Data are expressed as mean ± standard deviation (SD) for continuous variables and number (%) for categorical variables.

Characteristics	Data	Women	Men
Sex	37	22 (59.5%)	15 (40.5%)
Age (years)	62.5 ± 12.4 (CI 95%: 58.4–66.7)	61.2 ± 12.5 (CI 95%: 55.8–66.6)	64.7 ± 12.5 (CI 95%: 57.5–71.9)
Body max index (kg/m^2^)	24.6 ± 8.0 (CI 95%: 21.9–27.3)	27.4 ± 3.7 (CI 95%: 25.6–29.1)	26.0 ± 2.6 (CI 95%: 24.6–27.5)
Comorbidity			
Diabetes Mellitus Type 2	6 (16.2%)	3 (50%)	3 (50%)
Blood Hypertension	24 (64.9%)	8 (33.3%)	16 (66.7%)
Clinical data			
Visual analogue scale	7.4 ± 0.6 (CI 95%: 7.2–7.6)	7.3 ± 0.6 (CI 95%: 7.0–7.6)	7.5 ± 0.6 (CI 95%: 7.2–7.9)
Knee Injury and Osteoarthritis Outcome Score	45.9 ± 4.5 (CI 95%: 44.3–47.5)	46.4 ± 4.2 (CI 95%: 44.6–48.2)	45.3 ± 5.1 (CI 95%: 42.4–48.1)

**Table 2:** Pearson’s correlation coefficients between demographic and clinical variables (sex, age, body mass index [BMI], and pain intensity) in patients with knee osteoarthritis.

		Age vs. BMI	Age vs. Pain	BMI vs. Pain
All	Pearson’s correlation	−0.140	0.016	−0.189
	*p* value	0.408	0.927	0.264
Women	Pearson’s correlation	0.352	0.118	−0.256
	*p* value	0.140	0.600	0.291
Men	Pearson’s correlation	−0.348	−0.170	0.106
	*p* value	0.223	0.561	0.706

**Table 3:** Comparison of circulating cytokine and biomarker levels among patients with knee osteoarthritis using one-way analysis of variance (ANOVA).

Cytokines	F	*p* Value
TNFSF13	0.207	0.891
TNFSF13B	0.535	0.662
MMP1	0.027	0.994
MMP3	0.357	0.784
Pentraxin3	0.105	0.957
TNF_alfa	0.289	0.833
IL-6	0.447	0.721
IL-8	1.991	0.134
IL-10	1.570	0.215

**Table 4:** Sex-related differences in circulating matrix biomarker levels in patients with knee osteoarthritis, evaluated using the two-sample *t*-test. Data are expressed as mean ± standard deviation. TNF: Tumor necrosis factor; TNFSF13: TNF superfamily member 13 (APRIL); TNFSF13B: TNF superfamily member 13B (BAFF); MMP: matrix metalloprotease.

Cytokines	Men (n = 15)	Women (n = 22)	*p*-Value	Median Men	Median Women	Range Men	Range Women	IQR Men	IQR Women	Cohen’s d
TNFSF13	47,495.9 ± 19,794.2	53,104.9 ± 37,177.7	0.597	41,041.2	40,045.0	63,714.6	85,261.4	31,684.2	28,904.02	0.1883
TNFSF13B	3817.7 ± 2274.4	5388.9 ± 4770.2	0.244	4780.1	4828.1	5267.9	20,354.7	2926.1	2596.52	0.4205
MMP1	4539.4 ± 933.6	3984.3 ± 899.0	0.080	4226.1	3988.1	3280.78	3491.8	1322.2	1002.2	0.605
MMP3	48,492.2 ± 68,639.2	34,870.8 ± 5037.2	0.357	32,652.2	33,557.9	277,116.4	17,491.0	8799.4	7001.7	0.279
Pentraxin3	4870.9 ± 2599.3	5888.2 ± 2242.6	0.212	3927.4	5425.5	8200.5	6850.4	2924.3	3734.0	0.4191

**Table 5:** Sex-related differences in circulating inflammatory cytokines levels in patients with knee osteoarthritis, evaluated using the two-sample *t*-test. Data are expressed as mean ± standard deviation. TNF: Tumor necrosis factor. IL: Interleukin.

Cytokines	Men (n = 15)	Women (n = 22)	*p*-Value	Median Men	Median Women	Range Men	Range Women	IQR Men	IQR Women	Cohen’s d
TNF-alpha	50.5 ± 8.3	47.3 ± 8.4	0.500	52	46	29	34	9.5	8	0.3832
IL-6	42.9 ± 12.9	41.4 ± 7.1	0.468	46	41.5	49	33	15.5	9	0.1441
IL-8	62.1 ± 9.8	57.0 ± 7.0	0.0745	60	58.5	32	25	8.5	9	0.5989
IL-10	2.1 ± 0.8	2.3 ± 0.7	0.426	2.2	2.4	8	2.6	4	1.2	0.2661

**Table 6:** Pearson’s correlations between circulating inflammatory biomarkers and Knee Injury and Osteoarthritis Outcome Score (KOOS) scores in patients with knee osteoarthritis. The false discovery rate (FDR) was controlled at 5%, and statistical significance was defined as q < 0.05. CI: confidence interval; IL: interleukin; TNF: tumor necrosis factor; MMP: matrix metalloprotease; TNFSF13: TNF superfamily member 13 (APRIL); TNFSF13B: TNF superfamily member 13B (BAFF). * *p* < 0.05.

Biomarker	Pearson r	95% CI	*p* Value	q-Value (FDR)
IL-6	−0.337	−0.60 to −0.03	0.041	0.123
IL-10	0.449	0.15–0.69	0.005	0.045 *
TNF-α	−0.212	−0.50 to 0.09	0.148	0.266
IL-8	−0.187	−0.45 to 0.11	0.210	0.315
MMP-1	−0.164	−0.43 to 0.13	0.265	0.358
MMP-3	−0.198	−0.46 to 0.10	0.191	0.315
Pentraxin-3	−0.143	−0.42 to 0.12	0.310	0.387
TNFSF13	−0.129	−0.40 to 0.15	0.362	0.407
TNFSF13B	−0.154	−0.42 to 0.12	0.298	0.387

## Data Availability

The original contributions presented in the study are included in the article, further inquiries can be directed at the corresponding author. Data is available if requested.

## Supplementary Materials

The following supporting information can be downloaded at: https://www.mdpi.com/article/10.3390/jcm15093466/s1, Table S1: Clinical data and blood biomarkers of health patients; Tables S2: Clinical data and blood biomarkers of Knee Osteoarthritis patients.
